# Joint Reaction Forces Decrease Following Total Knee Arthroplasty up to 12‐Months Post‐Surgery

**DOI:** 10.1002/jor.70222

**Published:** 2026-05-10

**Authors:** Salindi P. Herath, David Hobbs, Dominic Thewlis, Christopher Wilson, Mark Taylor

**Affiliations:** ^1^ College of Science and Engineering, Medial Device Research Institute Flinders University Adelaide South Australia Australia; ^2^ Centre for Orthopaedic & Trauma Research Adelaide University Adelaide South Australia Australia; ^3^ College of Medicine and Public Health Flinders University Adelaide South Australia Australia

**Keywords:** joint reaction force, musculoskeletal modeling, total knee arthroplasty

## Abstract

Changes in mechanical loading following total knee arthroplasty (TKA) are a critical factor that is important to understand with respect to component fixation. The aims of this study were to estimate medial, lateral, and total knee joint contact forces preoperatively, and then at 3‐, 6‐, and 12‐months postoperatively. Joint reaction forces (JRFs) were estimated using an electromyography (EMG) informed musculoskeletal modeling workflow with models scaled using computed tomography (CT) scans and weightbearing X‐rays, for lower limb alignment. Joint loading peak values and impulses were estimated in 22 participants awaiting primary TKA for osteoarthritis (OA) (median age, range: 69, 49–80 years; median BMI, range: 33, 25–40 kg/m^2^) during level gait and sit‐to‐stand at each timepoint. Medial, lateral, and total JRF peak values and impulses reduced significantly postoperatively at 3‐months and did not return to preoperative values at 12‐months for either activity. During level gait, mean total JRF peak values reduced by 0.72 BW (*p* < 0.001) (medial mean difference: 0.39 BW, *p* < 0.001; lateral mean difference: 0.23 BW, *p* < 0.001) at 3‐months post‐surgery. A similar trend was observed in the sit‐to‐stand activity with a mean decrease of 0.44 BW in the peak total JRF at 3‐months (*p* < 0.001). Impulse values followed a similar trend to the peak values. Clinical relevance: People do not return to preoperative JRFs even at 12‐months post‐TKA, and this may be a contributing factor for previously reported decreases in BMD around femoral and tibial components.

## 1 Introduction

Total knee arthroplasty (TKA) is a routine procedure for reducing knee joint pain and improving function, with greater than 90% of surgeries worldwide conducted in people with knee osteoarthritis (OA) [1, 2, 3, 4]. One of the most common reasons for failure of primary TKA is aseptic loosening, which can be affected by bone resorption adjacent to the implant [5, 6, 7]. Following TKA, bone mineral density has been shown to decrease in the proximal tibia [8, 9, 10, 11, 12] and the distal femur [13, 14, 15, 16, 17, 18, 19, 20, 21], which can affect fixation of the components. Furthermore, regions closer to the component experienced greater bone loss compared to areas further distal [22], which may be clinically relevant with respect to implant fixation. Bone remodeling is known to be affected by the mechanical loads experienced in the area [23, 24]. A key step in understanding the mechanical factors that influence bone remodeling in the proximal tibia is the characterization of changes in joint loading, ideally at a compartment level, that occur following TKA.

Knee joint reaction forces (JRFs) can be measured in vivo using instrumented implants or estimated using musculoskeletal modeling. Although instrumented tibial components provide the most accurate measure of joint loading, they cannot be used to generalize, due to small cohorts, and can only measure postoperative JRFs when the pre–postoperative change in loading may be the factor influencing bone remodeling. Musculoskeletal modeling is a more widely available alternative to estimate changes in knee JRFs following TKA. The knee adduction moment has been used previously as a proxy measure of knee JRFs [25, 26], but the validity of this measure has been questioned [27, 28]. To date, two studies have investigated knee JRFs pre‐ and post‐TKA [29, 30] with both studies estimating muscle forces without electromyography (EMG), relying on optimally controlled muscle activation patterns that do not consider individual activation patterns or co‐contraction. Furthermore, people with knee OA exhibit variation in muscle co‐contraction [31] as well as activation duration and magnitude [32] compared to healthy controls that are not reflected in muscle force estimations in the absence of EMG [33, 34]. Finally, both Worsley et al. [30] and Hamandi et al. [29] only reported on the total knee JRF and did not estimate condyle‐specific loading.

The aim of this study was to characterize longitudinal changes to medial, lateral, and total knee JRFs following TKA during level gait and sit‐to‐stand utilizing state‐of‐the‐art musculoskeletal modeling methods. It was hypothesized that medial JRFs would decrease postoperatively, while lateral JRFs would increase as most people undergoing a TKA experience a lower limb alignment correction from varus to mechanically aligned [35]. Quantifying biomechanical changes that occur following TKA can further elucidate mechanisms of bone remodeling in the proximal tibia.

## 2 Materials and Methods

### 2.1 Participants

This study protocol was approved by the local hospital ethics committee (Southern Adelaide Local Health Network [2021/HRE00290]). Participants were considered for inclusion if awaiting TKA due to knee OA. Exclusion criteria were any diagnoses of neuromuscular disorders or if taking medication that altered bone remodeling. Written informed consent was obtained from all participants prior to surgery. Knee joint OA was graded by a consultant surgeon using Kellgren–Lawrence scores (K/L) on plain film radiography taken as part of preoperative planning.

### 2.2 Surgery

All participants received a cemented, cruciate retaining, and fixed bearing tibial component of the DePuy Attune (Depuy Synthes, Warsaw, Indiana, USA) or the Smith & Nephew Legion (Smith & Nephew, London, UK). All procedures were performed through a midline incision and medial parapatellar arthrotomy. All procedures were performed by a consultant surgeon or senior fellow in one elective arthroplasty center.

### 2.3 Patient‐Reported Outcome Measures

At each timepoint, participants completed three patient‐reported outcome measures (PROMs): Oxford Knee Score (OKS, Oxford University Innovation, UK), Knee injury and Osteoarthritis Outcomes Scores‐12 (KOOS‐12 [36]), and 5‐level EQ‐5D (EQ‐5D‐5L, EuroQol, the Netherlands). EQ‐5D‐5L index values are scored out of 1. OKS and each KOOS‐12 category were scored out of 48 and 100, respectively. Higher values correspond to better health outcomes for all PROMs.

### 2.4 Lower Limb Alignment

Lower limb alignment was assessed using the hip–knee–ankle angle [37], and the coronal plane alignment of the knee (CPAK) [38]. Preoperative alignment was measured from weightbearing X‐rays of the lower body. The virtual X‐ray tool on Simpleware Medical Software (Version 6.0; Synopsys Inc., Sunnyvale, USA) was used to determine postoperative alignment with surface models of the femur and tibia obtained from supine computed tomography (CT) scans and weightbearing postoperative X‐rays of the knee joint. These data were used to improve patient‐specificity of the musculoskeletal models by modifying the frontal plane orientation of the femur and tibia of the generic model prior to scaling skeletal anatomy (Section 2.6).

### 2.5 Motion Capture Experiment

Motion analysis was performed preoperatively, and then at 3‐, 6‐, and 12‐months postoperatively. Marker trajectories and ground reaction forces were captured using a 10‐camera Vicon system (Vicon Motion Systems, Oxford, UK) and four floor‐embedded force platforms (AMTI, MA, USA) at 100 and 1000 Hz, respectively. One investigator (S.P.H.) placed 46 surface markers on anatomical landmarks of the pelvis and lower limbs, and five rigid clusters consisting of four markers onto the thighs, lower legs, and sacrum.

Nine Delsys Trigno EMG sensors (Delsys, MA, USA) collected muscle excitations (2000 Hz) from the rectus femoris, vastus medialis, vastus lateralis, biceps femoris, semitendinosus, medial and lateral gastrocnemii, soleus, and tibialis anterior of the surgical leg using recommendations of the Surface Electromyography for the Non‐Invasive Assessment of Muscles guidelines [39].

Participants completed two activities of daily living at each assessment: level gait and sit‐to‐stand. During level gait, participants walked at a self‐selected pace across an 8‐m walkway. For the sit‐to‐stand task, participants began from a seated position on a 50 cm modified shower chair and stood up at their own speed, when able, following a verbal cue. Participants were instructed to sit with feet on two separate force platforms with arms crossed over the chest, if able.

### 2.6 Model Scaling

Knee JRFs were estimated from the experimental data using the modified Gait2392 model [40], consisting of 17 bodies and 92 musculotendon actuators with frontal plane motion locked [41].

MeshLab Software (v2022.02) was used to place 14 virtual markers (pelvis, femoral epicondyles, malleoli, and tibial epicondyles) onto surface models of the pelvis, femur, and tibia–fibula. Virtual marker positions were registered using MAPClient [42] with two principal components to capture skeletal dimensions, a Mahalanobis weighting of 0.1, and 0 mm of soft tissue padding to obtain scale factors by comparing marker positions between the morphed and generic model (Supplementary Data).

Optimal fiber and tendon slack lengths of muscle actuators were optimized using the muscle parameter optimizer MATLAB tool [43]. Muscle maximum isometric forces were personalized using muscle volume fractions based on participant mass [44, 45].

### 2.7 Data Processing

Marker trajectory and external load data were prepared using MotoNMS [46] in MATLAB R2019 (Mathworks, USA), which included a low‐pass filter (10Hz and 8 Hz, respectively) using a second‐order zero‐lag Butterworth filter. EMG signals were filtered with a second‐order bandpass filter (30 and 300 Hz), full‐wave rectified, low‐pass filtered (6 Hz), and normalized to the maximum value obtained from that session [47, 48].

Kinematics, kinetics, and muscle parameters were solved using OpenSim 3.3 [49]. To improve dynamic consistency between kinematics and external loads, the center of pressure was translated in the axial plane, after solving inverse kinematics, to match its position from the ankle joint center seen in the raw data.

Muscle forces were estimated using the Calibrated EMG‐informed Neuromusculoskeletal Modelling Toolbox (CEINMS) [34]. Processed EMG envelopes were mapped to 12 corresponding muscle‐tendon units. One calibrated model was generated per session using five randomly selected trials including both activities [47], aiming to optimize simulated knee joint sagittal plane moments and maximum medial and lateral knee JRF [50].

The sit‐to‐stand activity cycle was defined from when the trunk angular velocity reached 10% of its peak‐to‐peak value and ended when the vertical force remained within 1% of bodyweight [51].

Walking speed and cadence were calculated from level gait. Cycle time was calculated from both activities as the time spent during the stance phase for level gait and the time taken to complete standing for sit‐to‐stand.

Medial and lateral knee JRFs were estimated with the EMG‐informed muscle forces and frontal plane kinetics [52]. Total knee JRF was the summation of the medial and lateral forces.

Three trials per activity were selected from the beginning, middle, and end of the session to capture variability that could occur during the session. Minimum and maximum knee flexion angles and range of motion (ROM) were calculated from kinematics as well as peak and impulse values for medial, lateral, and total JRFs. Impulses were calculated as the area under the curve during the activity cycle to provide an estimation of the total energy imparted onto the joint.

### 2.8 Statistical Analysis

Comparisons between timepoints were conducted using a linear mixed effects model analysis with random intercept and heterogeneous first‐order autoregressive covariance structure to account for missing values (SPSS 27, IBM SPSS Statistics, USA). Pairwise comparisons between timepoints were conducted with a least significant difference correction [53]. Statistical parametric mapping (SPM) paired two‐way *t*‐tests were conducted on kinematics and JRFs. Statistical significance was defined at *p* < 0.05.

## 3 Results

### 3.1 Participants

**Figure 1:**
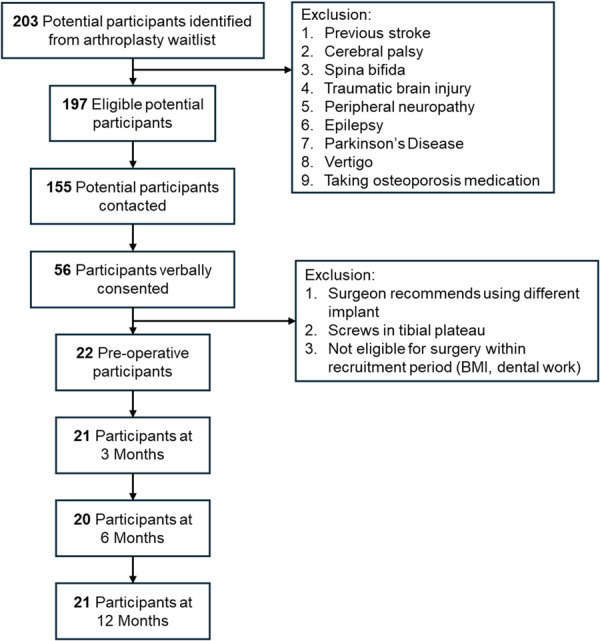
Participant recruitment flow chart with inclusion and exclusion criteria as well as the number of participants at each follow‐up timepoint. BMI, body mass index.

Twenty‐two participants (12 female) were enrolled in this study with a mean age of 69 years and a mean BMI of 33 kg/m^2^ (Figure 1, Table 1). Median K/L grade was 3 (range: 2–4). Participants missed follow‐up assessments at 3‐months (*n* = 1), 6‐months (*n* = 2), and 12‐months (*n* = 1). Twenty participants received the DePuy Attune (Depuy Synthes, Warsaw, Indiana, USA) with one participant receiving a revision tibial component with a longer stem. Two people received the Smith & Nephew Legion (Smith & Nephew, London, UK).

**Table 1:** Participant details and patient‐reported outcome measure (PROM) responses preoperatively (pre‐op), and 3‐, 6‐, and 12‐months post‐surgery.

Parameters	Pre‐op	3 months	6 months	12 months
*N* (*n* female)	22 (12)	21 (11)	20 (11)	21 (11)
Age at surgery median (range) years	69 (49–80)			
BMI (range) (kg/m^2^)	33 (25–40)	34 (26–40)	33 (25–41)	35 (25–42)
Body mass (range) (kg)	91 (68–127)	91 (68–129)	91 (70–130)	96 (71–135)
K/L score median (range)	3 (2–4)			
KOOS‐12 pain mean (SD)	36 (15)	75 (22)	76 (19)	80 (22)
KOOS‐12 function mean (SD)	43 (13)	78 (19)	75 (19)	80 (17)
KOOS‐12 quality of life mean (SD)	23 (16)	61 (25)	66 (23)	70 (26)
OKS mean (SD)	21 (6)	34 (8)	37 (8)	39 (7)
EQ‐5D‐5L index value mean (SD)	0.65 (0.24)	0.90 (0.10)	0.91 (0.10)	0.94 (0.06)

Abbreviations: BMI, body mass index; EQ‐5D‐5L, 5‐level EQ‐5D; K/L, Kellgren–Lawrence; KOOS, knee injury and osteoarthritis score; OKS, Oxford knee score; SD, standard deviation.

On average, preoperative PROMs were scored less than 50% of their respective maximum values except for EQ‐5D‐5L (65%) (Table 1). Participants reported a reduction in pain, an increase in function, and a higher general quality of life at the 3‐month timepoint, with the best self‐reported health outcomes at 12‐months postoperation.

Preoperatively, most participants (*n* = 16) were varus aligned based on the HKA angle (median HKA angle of 172°). Postoperatively, all participants were closer to mechanical alignment (Table 2).

**Table 2:** Lower limb alignment pre‐ and postoperation classified using hip–knee–ankle (HKA) angle and coronal plane alignment of the knee (CPAK) types.

Timepoint and parameter	*n*	HKA angle median (range)
Preoperative		
Varus (HKA angle)	16	172 (166–176)
Neutral (HKA angle)	2	178 (178–179)
Valgus (HKA angle)	4	191 (184–202)
Varus apex distal (CPAK‐I)	8	
Neutral apex distal (CPAK‐II)	1	
Varus neutral (CPAK‐IV)	8	
Neutral (CPAK‐V)	1	
Valgus neutral (CPAK‐VI)	3	
Valgus apex proximal (CPAK‐IX)	1	
Postoperative		
Varus (HKA angle)	8	175 (169–177)
Neutral (HKA angle)	9	178 (177–182)
Valgus (HKA angle)	2	185 (183–187)
Varus apex distal (CPAK‐I)	7	
Neutral apex distal (CPAK‐II)	4	
Varus neutral (CPAK‐IV)	3	
Neutral (CPAK‐V)	3	
Valgus neutral (CPAK‐VI)	2	
Valgus apex proximal (CPAK‐IX)	0	

### 3.2 Spatiotemporal Parameters

**Figure 2:**
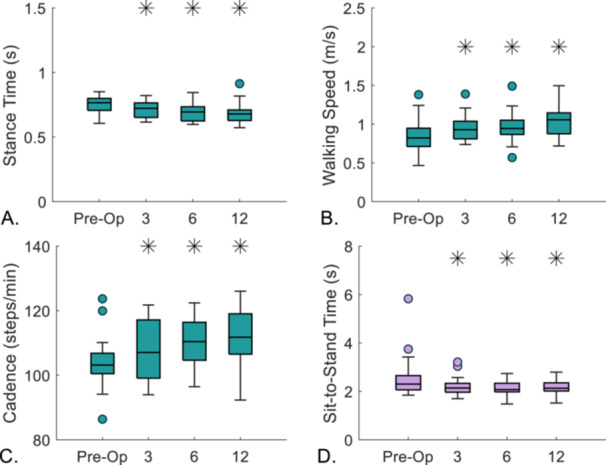
Box and whisker plots of spatiotemporal parameters for level gait and sit‐to‐stand. (A) Stance time for level gait (s). (B) Walking speed (m/s) for level gait. (C) Cadence (steps/min). (D) Cycle time for sit‐to‐stand (s). *indicates significance (*p* < 0.05) compared to preoperative timepoint. The box bottom and top show 25th and 75th percentiles with the median value across each box. Whiskers above and below the boxes display the maximum and minimum values. Outliers, indicated by filled circles, are calculated as 1.5 times the interquartile range.

At 3 months postoperation, walking speed increased (Δ = 0.08 m/s, *p* = 0.006) with a decrease in stance time (Δ = 0.04 s, *p* = 0.001) and an increase in cadence (Δ = 4 steps/min, *p* = 0.011) during level gait and continued to improve at postoperative timepoints (Figure 2). Cycle time for sit‐to‐stand decreased significantly at 3‐months postoperation (Δ = 0.31 s, *p* = 0.023) and did not change significantly during follow‐up timepoints (Supplementary Data).

### 3.3 Knee Kinematic Parameters

Knee flexion angles did not change dramatically between preoperative and postoperative timepoints for either level gait or sit‐to‐stand (Supplementary Data).

### 3.4 Joint Reaction Forces

**Figure 3:**
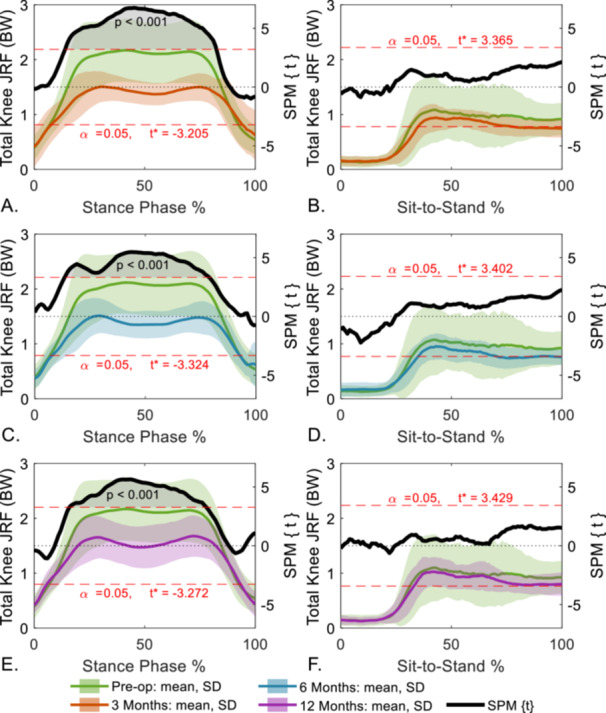
Statistical parametric mapping (SPM) paired two‐tailed *t*‐test for comparing knee joint reaction force (JRF) values between pre‐operation and (A, B) 3‐months post‐surgery, (C, D) 6‐months post‐surgery, and (E, F) 12‐months post‐surgery for level gait (left) and sit‐to‐stand (right). Significance was set at *α* = 0.05 for all comparisons with *t*‐statistic displayed on the right vertical axes in all plots.

Total postoperative JRF curves exhibited more pronounced double peak curves compared to preoperatively during level gait (Figure 3). SPM results indicated a statistically significantly greater preoperative total JRF between 20% and 80% of the gait cycle compared to 3‐months postoperation (Figure 3).

**Figure 4:**
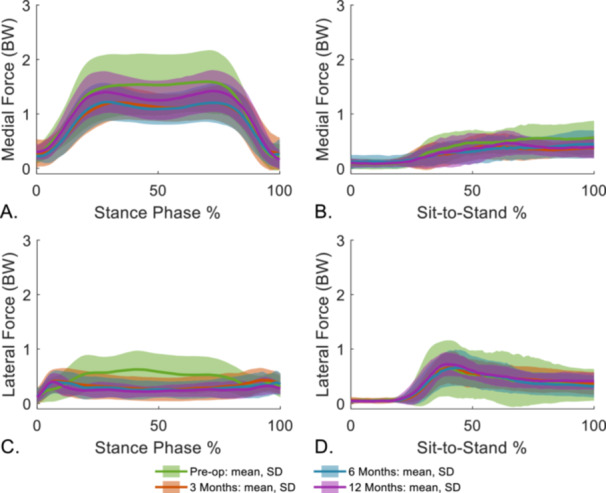
Medial, lateral, and total joint reaction forces (JRF) for level gait (left) and sit‐to‐stand (right) normalized to the activity cycle. Cohort mean and standard deviations (SDs) are presented for preoperative and three follow‐up timepoints. BW, bodyweight.

During sit‐to‐stand, lateral JRF was greater than medial at all timepoints (Figure 4). Preoperatively, rapid fluctuations were present that were especially prominent in the total JRF, which lessened postoperatively. Variability between participants decreased at postoperative timepoints. SPM did not reveal any significant differences between pre‐ to postoperation (Figure 3).

#### 3.4.1 Peak Values

**Figure 5:**
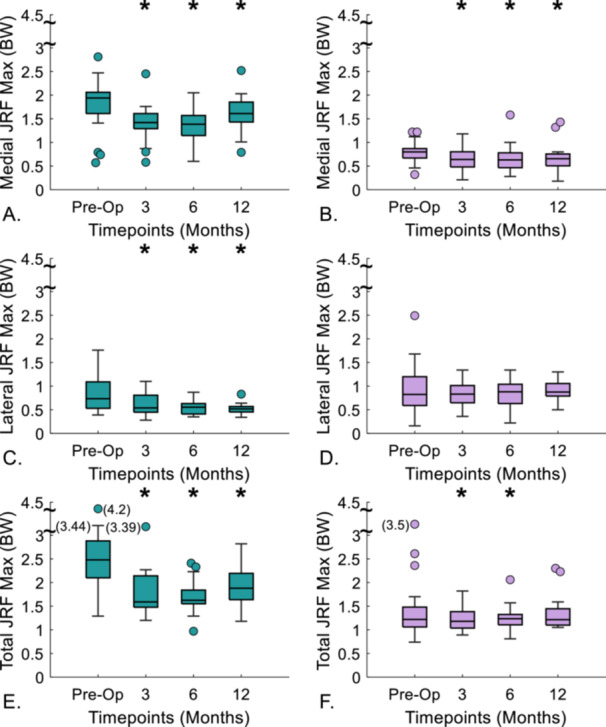
Peak knee joint reaction forces (JRF) for walking (left) and sit‐to‐stand (right) preoperatively and then at three follow‐up timepoints (*indicates significant *p* < 0.05) difference compared to preoperatively). BW, bodyweight. Box bottom and top show 25th and 75th percentiles with the median value across each box. Whiskers above and below the boxes display the maximum and minimum values. Outliers, indicated by filled circles, are calculated as 1.5 times the interquartile range.

Peak values for JRF were greatest preoperatively for medial, lateral, and the total knee load for level gait (1.81, 0.84, and 2.52 BW, respectively) (Figure 5, Supplementary Data). During level gait, medial JRF peak values decreased significantly at 3‐months by 0.39 BW (*p* < 0.001), and continued to decrease at 6‐months by 0.05 BW (*p* = 0.506 compared to 3‐month), and then increased at 12‐months by 0.24 BW (*p* < 0.001 compared to 6‐month) (Figure 5). Lateral JRF peak values during the stance phase of level gait decreased at 3‐months post‐surgery by 0.23 BW (*p* < 0.001) and did not change significantly at 6‐ and 12‐months (*p* = 0.432, *p* = 0.577, respectively, compared to the previous follow‐up timepoint). Maximum total JRF values followed a similar trend to the peak medial JRF values.

During sit‐to‐stand, peak values for medial, lateral, and total JRF were greatest preoperatively (0.96, 0.88, 1.84 BW) (Figure 5, Supplementary Data). At 3 months postoperation, peak medial and total JRF values decreased significantly by 0.30 and 0.44 BW, respectively (*p* < 0.001 for both JRFs). Lateral JRF peak values also decreased at 3‐months post‐surgery, although not reaching statistical significance (Δ = 0.10 BW, *p* = 0.376) (Figure 5). Peak values were the lowest 6‐months postoperatively for medial, lateral, and total JRFs with a significant decrease in lateral JRF peak value at 6‐months compared to 3‐months post‐surgery (0.03 BW, *p* = 0.030). At 12‐months post‐surgery, lateral peak values increased more than medial compared to the previous timepoint (Δ = 0.11 BW vs. 0.01 BW). No statistically significant differences were seen between preoperative and 12 month postoperative timepoints for peak total JRF values (*p* = 0.148) (Figure 5).

#### 3.4.2 Impulses

**Figure 6:**
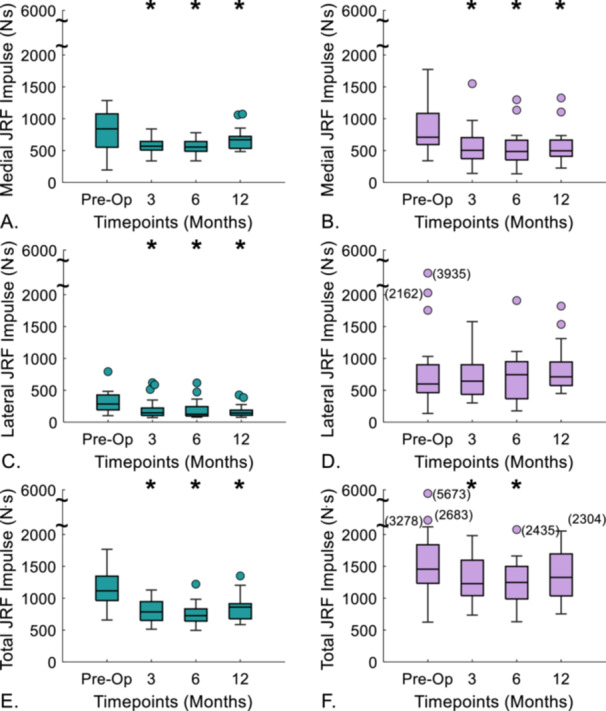
Knee joint reaction force (JRF) impulses for (A–C) level gait and (D–F) sit‐to‐stand preoperatively and then at three follow‐up timepoints. *indicates significance (*p* < 0.05) compared to preoperative timepoint. The box bottom and top show 25th and 75th percentiles with the median value across each box. Whiskers above and below the boxes display the maximum and minimum values. Outliers, indicated by filled circles, are calculated as 1.5 times the interquartile range.

During level gait, JRF impulses were greatest preoperatively for medial, lateral, and total load (813, 316, 1129 N⋅s, respectively) (Figure 6, Supplementary Data). Medial and lateral JRF impulses followed a similar trend to their respective peak values, with the lowest medial and total impulse at 6‐months (556 N⋅s, 747 N⋅s, respectively), and the lowest lateral impulse at 12 months (170 N⋅s). Medial and total impulse also increased significantly between 6 and 12 months (Δ = 113 N⋅s, *p* < 0.001 and Δ = 92 N⋅s, *p* = 0.014, respectively) (Supplementary Data).

Medial, lateral, and total JRF impulses were highest preoperatively for sit‐to‐stand (701, 1430, 2131 N⋅s, respectively) (Figure 6). Medial and total JRF impulses for sit‐to‐stand followed a similar trend to their respective peak values with the lowest values at 6‐months post‐surgery (538 and 1247 N⋅s) and no significant difference in total impulse between preoperative and 12‐month postoperative timepoints (*p* = 0.084) (Supplementary Data). Lateral impulse was the lowest at 6‐months post‐surgery with no significant difference compared to 12‐months (*p* = 0.153).

## 4 Discussion

This study aimed to determine if joint loading changes during activities of daily living up to 12 months following a TKA using musculoskeletal modeling. The findings of this study were that there is a statistically significant decrease in peak loads and impulses at 3‐months post‐TKA that begin to increase following 6‐months post‐surgery, but do not return to preoperative levels at 12‐months following surgery for either level gait or sit‐to‐stand.

Overall, participant patient‐reported outcomes showed a strong positive improvement following TKA. At the first follow‐up timepoint, greater than 70% of participants reported a decrease in pain and an increase in function that exceeded the minimal clinically important difference (MCID) of 13.5 and 15.2 for the KOOS‐12 [54] and the OKS (5.0 and 4.3, respectively) [55]. Metrics for quality of life and overall health also improved beyond the MCID of 8.0 for the KOOS‐12 [54] and 0.2 for the EQ‐5D‐5L [56] in greater than 50% of participants. Participants who did not meet the MCID for EQ‐5D‐5L (*n* = 9, 12, and 10 for 3‐, 6‐, and 12‐months, respectively) had an index value greater than 0.77 preoperatively.

On average, postoperatively, participants were able to complete tasks faster with improvements sustained at 12‐months. Preoperative expectations on postoperative knee function outcomes are a predictor of patient satisfaction [57], and the ability to complete activities of daily living, such as walking and rising from a seated position, is related to overall perception of knee function [58]. Changes in knee kinematics, however, were minimal with a slight increase in knee extension at heel strike during level gait and standing at the end of sit‐to‐stand, coinciding with previous literature and healthy knee kinematics [31, 32, 59, 60].

The JRF time‐histories in this study were qualitatively similar in shape and magnitude to instrumented data [61, 62] with a medially biased JRF during level gait and a lateral bias during sit‐to‐stand [63, 64, 65]. The peak total loads found in this study are within the range of in vivo values measured up to 6‐months post‐surgery for both level gait (1.17–2.47 BW) [61, 62] and sit‐to‐stand (1.56–2.13 BW) [62, 66]. For level gait, in vivo data showed a decrease in peak total JRF between 3 weeks and 5.5 months postoperation of 0.03 BW [62] similar to this study between the first two postoperative timepoints. Contrarily, previous literature that estimated JRFs using musculoskeletal modeling reported a 0.3 BW increase in peak total joint loading at 6‐months or a 0.8 BW increase in average stance phase JRF at 3‐months compared to preoperatively [29, 30]. However, the peak total forces reported in previous literature from musculoskeletal modeling (3.5 BW at 6‐months [30] during level gait and 4.64 BW at 13 months post‐surgery for sit‐to‐stand [67]) were outside the range measured from instrumented implants, which could be affecting the estimated changes post‐surgery. Differences between musculoskeletal modeling estimations of JRF and those measured from instrumented data are evident [68] due in part to model scaling [69], errors in marker position, and inaccurate muscle force estimations [33]. Differences are also, however, present between individuals as observed from instrumented data with one individual exhibiting a total peak load of 2.13 BW at 6‐months, whilst another 1.79 BW at 7.5‐months [62].

From 3‐months post‐surgery, total JRF profiles became more similar between participants. Participants had prominent double‐peak JRF curves during level gait following surgery, a common feature of nonpathological gait [26]. JRF curves during sit‐to‐stand have noise present in the estimations, which are not present in the flexion/extension knee kinematics for the motion. We observed that noise in the JRF estimation may result from inaccurate secondary knee motions, which may create erroneous moment arms. In the MSK model used in this study, knee joint translations and internal/external rotation, prescribed as a function of knee flexion/extension, may not be representative of a pathological or an implanted knee.

Up to 6‐months following surgery, the peak medial JRF decreased during level gait and sit‐to‐stand with the largest decrease at 3‐months following surgery between consecutive timepoints, in line with the authors' hypothesis. During level gait, there was a significant increase in the peak medial force between 6‐ and 12‐months following surgery, but the final peak force remained significantly lower than preoperative levels. Peak medial load increased during the sit‐to‐stand activity as well; however, this change did not reach statistical significance. Contrary to the hypothesis, peak lateral JRFs followed a similar trend to the medial; however, no significant changes were seen between consecutive timepoints after 3‐months post‐surgery for either activity. Medial and lateral impulses followed a similar pattern to the peak loads for both activities, which is in part due to the decrease in the time spent completing the activity. However, considering the decrease in peak load, the reduction in the impulse is not solely due to the decrease in activity cycle time. Future studies should consider changes to load transfer; however, the reduction in the magnitude of joint loading corroborates the reductions in bone density reported in previous literature.

Peak medial loads and impulses were higher preoperatively than postoperatively, which could be due to this cohort being primarily varus aligned leading to higher medial loading [63]. Postoperative lower limb alignment was closer to mechanical alignment thus contributing to a reduction in medial JRF peak and impulses. People with OA have also been reported to present with increased joint loading [70], greater knee adduction moments, and increased muscle activation [26, 31, 32]. Longitudinal studies have also reported a reduction in the adduction moment following TKA [71], which corroborates with a decrease in knee loading postoperatively. A shift in lower limb alignment from varus to closer to mechanical alignment following surgery seen in a majority of the cohort could be contributing to the reduction in medial knee load and by extension the total knee load since a majority of the total knee load is comprised of medial loading for level gait [72]. However, a reduction in lateral load is seen, even when excluding preoperatively valgus participants (0.80–0.62 BW at 3‐months post‐surgery), indicating that joint loads appear to decrease postoperatively irrespective of lower limb alignment.

One of the main limitations of this study is that musculoskeletal modeling estimations of JRF are limited in accuracy, in part due to scaling and muscle force estimations. Although the skeletal dimensions of the models were determined from medical imaging, specific bony geometries were not captured in this process, which can affect secondary knee kinematics and moment arms. Similarly, the knee joint model used in this study consisted of a hinge joint that acts at the center of the proximal tibia, which is physiologically inaccurate.

Muscle forces were estimated using EMG to improve subject‐specificity; however, data were not collected for all the muscles of the lower limb leading to a partially mathematically optimized calculation of muscle forces. EMG data were normalized based on the maximum voltages seen during a trial session, as is suggested in the absence of maximum voluntary isometric contractions for this cohort [73]. However, this reference point for normalization does not have high repeatability across different timepoints. We did not measure maximum voluntary isometric contractions to avoid excessive pain or discomfort to the participants. Furthermore, no single standardized muscle test exists for obtaining maximum voluntary isometric contractions therefore requiring multiple tests for accuracy, which could increase participant pain and discomfort. Another limitation of this study is the sample size of the participants, which may not allow for generalization of the results across the population.

Positive perception of outcomes following surgery was demonstrated in spatiotemporal parameters and JRF curves that trend towards improved knee function. JRF peak values and impulses decreased following surgery during both level gait and sit‐to‐stand with reductions retained at 12‐months post‐surgery. A statistically significant increase in knee loading during level gait is seen between 6‐ and 12‐months post‐TKA for level gait, which indicates that knee loads may return to preoperative levels at a later follow‐up time than was included in this study. Sustained reductions in joint loading following a TKA may be detrimental to implant fixation, and future studies should investigate the relationship between bone remodeling and JRF changes.

## Author Contributions

**Salindi P. Herath:** substantial contribution to research design, acquisition, analysis, interpretation of results, and drafting of paper. **David Hobbs:** substantial contribution to research design, acquisition, analysis, interpretation of results, and revising the paper critically. **Dominic Thewlis:** substantial contribution to research design, acquisition, analysis, interpretation of results, and revising the paper critically. **Christopher Wilson:** substantial contribution to acquisition of results. **Mark Taylor:** funding, substantial contribution to research design, acquisition, analysis, interpretation of results, revising the paper critically, and approval of the submitted and final versions.

## Supporting information

Supporting File

## Data Availability

The data that support the findings of this study are available from the corresponding author upon reasonable request.
