# Supplementary material for: Joint Reaction Forces Decrease Following Total Knee Arthroplasty up to 12‐Months Post‐Surgery
Source: J Orthop Res. 2026 May 10;44:e70222. doi: 10.1002/jor.70222 (PMC13157651; doi:10.1002/jor.70222)
Supplement: Supplementary file 1 — Supporting File [file JOR-44-0-s001.docx]

**Table 1.** Scale factors for dimensions of the pelvis, thigh, and shank for linear and statistical shape modelling (SSM) scaling.

| **Parameter** | **Marker-SSM, CT-SSM, Alignment Scaling** |
| --- | --- |
| **HJC** |  |
| Location | Centre of sphere fit to femoral head |
| **KJC** |  |
| Location | Midpoint of femoral epicondyles |
| **AJC** |  |
| Location | Midpoint of malleoli markers |
| **Pelvis** |  |
| Depth | Distance from midpoint between PSIS markers to midpoint between ASIS markers |
| Width | Distance between ASIS markers |
| Height | Average of depth and height scale factors |
| **Thigh** |  |
| Height | Distance between HJC and KJC |
| Width | Distance between femoral epicondyles |
| Depth | Average of width and height scale factors |
| **Calf** |  |
| Width | Distance between malleoli markers |
| Height | Distance from midpoint between tibial condyles to AJC |
| Depth | Average of depth and height scale factors |

ASIS: anterior superior iliac spine

AJC: ankle joint centre

CT: computed tomography

HJC: hip joint centre

KJC: knee joint centre.

PSIS: posterior superior iliac spine

**Table 2.** Level gait mean and standard deviation (SD) of surgical leg knee kinematics at pre-operation (pre-op) and follow-up timepoints. Positive values indicate extension. *p* - values are comparisons to pre-op.

| **Kinematic Parameters** | | **Pre-Op** | **3 Months** | **6 Months** | **12 Months** |
| --- | --- | --- | --- | --- | --- |
| **Level gait** |  |  |  |  |  |
| Minimum flexion | Mean (°) (SD) | 0 (10) | 0 (8) | -2 (7) | -4 (7) |
|  | *p* – value |  | 0.462 | 0.178 | 0.050 |
| Maximum flexion | Mean (°) (SD) | 32 (9) | 33 (12) | 31 (13) | 33 (12) |
|  | *p* – value |  | 0.649 | 0.929 | 0.711 |
| ROM | Mean (°) (SD) | 31 (8) | 35 (7) | 37 (5) | 38 (5) |
|  | *p* – value |  | **0.015** | **0.002** | **0.001** |
| **Sit-to-Stand** |  |  |  |  |  |
| Minimum flexion | Mean (°) (SD) | 6 (12) | 3 (10) | 2 (10) | 0 (9) |
|  | *p* – value |  | 0.242 | 0.118 | **0.032** |
| Maximum flexion | Mean (°) (SD) | 86 (8) | 79 (20) | 72 (31) | 82 (20) |
|  | *p* – value |  | **0.021** | 0.087 | 0.590 |
| ROM | Mean (°) (SD) | 81 (15) | 76 (21) | 70 (31) | 81 (22) |
|  | *p* – value |  | 0.653 | 0.907 | 0.200 |

ROM: range of motion

SD: standard deviation

|  |  |
| --- | --- |
|  |  |
|  |  |
|  | |
| Figure 1. Statistical parametric mapping (SPM) paired two-tailed t-test for comparing knee flexion kinematics between pre-operation and A-B. 3-months post-operation, C-D. 6-months post-operation, and E-F. 12-months post-operation for level gait (left) and sit-to-stand (right). Knee flexion angle is displayed on the left axis and SPM t statistic is displayed on the right vertical axis. | |

Table 3. Spatiotemporal parameters for level gait and sit-to-stand with *p-*values for comparison to preoperative values.

| **Spatiotemporal Parameters** |  | **Pre-Op** | **3 Months** | **6 Months** | **12 Months** |
| --- | --- | --- | --- | --- | --- |
| Stance time (s) | Mean (SD) | 0.75 (0.07) | 0.71 (0.06) | 0.69 (0.07) | 0.68 (0.08) |
|  | *p-*value |  | 0.001 | <0.001 | <0.001 |
| Sit-to-stand time (s) | Mean (SD) | 2.60 (0.89) | 2.19 (0.37) | 2.14 (0.28) | 2.17 (0.29) |
|  | *p-*value |  | 0.023 | 0.016 | 0.025 |
| Cadence (steps/min) | Mean (SD) | 104 (8) | 108 (9) | 110 (7) | 112 (9) |
|  | *p-*value |  | 0.011 | 0.001 | <0.001 |
| Walking speed (m/s) | Mean (SD) | 0.86 (0.22) | 0.94 (0.18) | 0.97 (0.20) | 1.03 (0.18) |
|  | *p-*value |  | 0.006 | 0.001 | <0.001 |

SD: standard deviation

Table 4. Medial, lateral, and total joint reaction force (JRF) mean peak values with standard deviation (SD) for level gait and sit-to-stand.

|  | Mean JRF Peak (BW) (SD) | | | | | | | |
| --- | --- | --- | --- | --- | --- | --- | --- | --- |
|  | **Level Gait** | | | | **Sit-to-Stand** | | | |
|  | Pre-op | 3 Months | 6 Months | 12 Months | Pre-op | 3 Months | 6 Months | 12 Months |
| **Medial JRF** | 1.81 (0.55) | 1.42 (0.38) | 1.37 (0.35) | 1.61 (0.37) | 0.96 (0.39) | 0.62 (0.35) | 0.60 (0.32) | 0.61 (0.30) |
| *p*-value ^a^ |  | **< 0.001** | **< 0.001** | **0.048** |  | **<0.001** | **0.001** | **0.001** |
| *p*-value ^b^ |  |  | 0.506 | **< 0.001** |  |  | 0.992 | 0.69 |
| **Lateral JRF** | 0.84 (0.36) | 0.61 (0.23) | 0.55 (0.15) | 0.52 (0.11) | 0.88 (0.76) | 0.78 (0.28) | 0.75 (0.35) | 0.86 (0.27) |
| *p*-value ^a^ |  | **< 0.001** | **< 0.001** | **< 0.001** |  | 0.376 | 0.594 | 0.759 |
| *p*-value ^b^ |  |  | 0.432 | 0.577 |  |  | **0.03** | 0.771 |
| **Total JRF** | 2.52 (0.65) | 1.80 (0.49) | 1.70 (0.34) | 1.91 (0.39) | 1.84 (0.93) | 1.40 (0.29) | 1.35 (0.27) | 1.47 (0.38) |
| *p*-value ^a^ |  | **< 0.001** | **< 0.001** | **< 0.001** |  | **0.016** | **0.04** | 0.148 |
| *p*-value ^b^ |  |  | 0.515 | **0.001** |  |  | 0.987 | 0.404 |

*p*-value ^a^: compared to pre-operation, *p*-value ^b^: compared to previous follow-up timepoint.

BW: bodyweight

Table 5. Medial, lateral, and total joint reaction force (JRF) mean impulses with standard deviation (SD) for level gait and sit-to-stand.

|  | Mean JRF Impulse (N·s) (SD) | | | | | | | |
| --- | --- | --- | --- | --- | --- | --- | --- | --- |
|  | **Level Gait** | | | | **Sit-to-Stand** | | | |
|  | Pre-op | 3 Months | 6 Months | 12 Months | Pre-op | 3 Months | 6 Months | 12 Months |
| **Medial JRF** | 813 (310) | 585 (129) | 556 (111) | 669 (165) | 701 (352) | 866 (387) | 538 (282) | 560 (270) |
| *p*-value ^a^ |  | **< 0.001** | **< 0.001** | **0.003** |  | **< 0.001** | **< 0.001** | **0.001** |
| *p*-value ^b^ |  |  | 0.241 | **< 0.001** |  |  | 0.362 | 0.286 |
| **Lateral JRF** | 316 (161) | 212 (165) | 191 (173) | 170 (95) | 1430 (717) | 854 (838) | 708 (422) | 832 (371) |
| *p*-value ^a^ |  | **< 0.001** | **< 0.001** | **< 0.001** |  | 0.259 | 0.427 | 0.960 |
| *p*-value ^b^ |  |  | 0.355 | 0.268 |  |  | 0.689 | 0.153 |
| **Total JRF** | 1129 (333) | 797 (129) | 747 (173) | 839 (203) | 2131 (869) | 1721 (1057) | 1247 (411) | 1393 (446) |
| *p*-value ^a^ |  | **< 0.001** | **< 0.001** | **< 0.001** |  | **0.002** | **0.006** | 0.084 |
| *p*-value ^b^ |  |  | 0.099 | **0.014** |  |  | 0.609 | 0.093 |

*p*-value ^a^: compared to pre-operation (pre-op)

*p*-value ^b^: compared to previous follow-up timepoint.

N: Newtons, s: seconds

|  |  | |
| --- | --- | --- |
|  |  | |
|  |  |  |
|  | |  |
| Figure 2. Statistical parametric mapping (SPM) results for vertical and medial-lateral components of ground reaction force (GRF) at pre-operation, and 3-months post-operation for level gait (left) and sit-to-stand (right) in the pre-operatively varus subgroup. Medial-lateral GRF moment arms with respect to the knee joint centre (E.-F.) are also presented with medial being positive and lateral being negative. | | |

|  |  | |
| --- | --- | --- |
|  |  | |
|  |  |  |
|  |  |  |
|  |  |  |
|  | |  |
| Figure 3. Pre- and 3 Months post-operative electromyography (EMG) envelopes for A. Rectus femoris, B. Vastus medialis, C. Vastus lateralis, D. Biceps femoris, E. Semitendinosus, F. Medial gastrocnemius, G. Lateral gastrocnemius, H. Soleus, I. Tibialis anterior for the surgical leg. All results are presented for stance phase of level gait. | | |

|  |  |
| --- | --- |
|  |  |
|  |  |
|  |  |
|  |  |
|  | |
| Figure 4. Pre- and 3 Months post-operative electromyography (EMG) envelopes for A. Rectus femoris, B. Vastus medialis, C. vastus lateralis, D. Biceps femoris, E. Semitendinosus, F. Medial gastrocnemius, G. Lateral gastrocnemius, H. Soleus, I. Tibialis anterior for the surgical leg. All results are presented for the activity cycle during sit-to-stand. | |

|  |  | |
| --- | --- | --- |
|  |  | |
|  | |  |
| Figure 5. Pre- and 3 Months post-operative external (ext.) knee flexion (A., B.) and adduction (C., D.) moment. Negative values indicate extension and abduction moments. Results are presented for stance phase of level gait (left) and sit-to-stand (right). All moments are normalized to participants’ weight and height. | | |
